# Cognitive Differences in the Older Adults Living in the General Community: Gender and Mental Occupational State Study

**DOI:** 10.3390/ijerph18063106

**Published:** 2021-03-17

**Authors:** Estela Calatayud, Carlos Salavera, Isabel Gómez-Soria

**Affiliations:** 1 Department of Physiatry and Nursing, Health Sciences Faculty, University of Zaragoza, 50009 Zaragoza, Spain; estelacs@unizar.es (E.C.); isabelgs@unizar.es (I.G.-S.); 2 Department of Psychology and Sociology, Education Faculty, University of Zaragoza, 50009 Zaragoza, Spain

**Keywords:** cognitive impairment, gender difference, subjective memory complaints, cardiovascular risk factors, occupational level

## Abstract

Older adults are particularly vulnerable to cognitive impairment with age, and gender differences are remarkable. However, there is very little evidence to identify both baseline cognitive and occupational gender differences prior to older adults’ retirement to design more efficient personalized cognitive interventions. This descriptive observational study examined gender differences in initial cognitive performance in 367 older adults with subjective memory complaints from a primary healthcare center in Zaragoza (Spain). To evaluate initial cognitive performance, the Spanish version of the Mini-Mental State Examination (MEC-35) and the set test were used to measure verbal fluency. Sociodemographic and clinical characteristics were evaluated, and cognitive and occupational differences were analyzed per gender. Men had higher educational and occupational levels, were older and more of them were married (*p* < 0.001) than women. Regarding cardiovascular risk factors, diabetes and cerebrovascular accidents were more frequent in women, while hypercholesterolemia and obesity were more frequent in men (*p* < 0.001). High blood pressure was more frequent in women, but not significantly so (*p* = 0.639). Global cognition was higher in men (*p* < 0.001) for attention, calculation, and language (*p* < 0.001). Verbal fluency was higher in women, but the difference was not statistically significant (*p* = 0.105). These results could be generalized to other health centers in the province and other Spanish autonomous communities as their sociodemographic variables are similar. Individualized interventions that adapt to gender, cognitive and initial occupational performance should be developed and adapted to elderly populations living in the general community to maintain their cognitive capacity and prevent their cognitive impairment and the social health costs this would imply.

## 1. Introduction

Aging is a complex, dynamic, continuous, and irreversible process that affects all living beings whose changes intensify with age [1]. Between 2015 and 2050, the percentage of people over the age of 60 will almost doubl from 12% to 22% according to the WHO [2].

As the world population ages and life expectancy prolongs, cognitive changes will present formidable challenges in the healthcare, social, and financial domains [3,4,5]. In the elderly population, “successful” aging depends on maintaining optimal cognitive brain performance [6]. Although a certain degree of foreseeable brain impairment occurs with aging, significant cognitive impairment is not unavoidable and can be prevented [4,7] by environmental enrichment and cognitive training [8,9].

The cognitive functions most vulnerable to aging depend on the speed and efficiency of cognitive processing, such as working memory, executive tasks, and attention. Nevertheless, visual perception, language, and gnosis are later affected [6]. Mnesic dysfunction has been the most studied because it is considered the most widespread cognitive alteration to occur during normal and pathologic aging [10].

Subjective cognitive impairment (SCD) is defined as a self-perceived cognitive impairment among cognitively normal individuals [11]. Several studies have suggested that SCD may be associated with an increased risk of mild cognitive impairment (MCI) [12] or dementia [11] due to Alzheimer’s disease (AD) [12]. It has been observed that subjective memory complaints (SMCs) are cognitive symptoms which, despite not being presented with objective cognitive alterations [13], are worth taking seriously as a possible early sign of impairment that could lead to dementia [14]. Besides, SMCs are related to a more marked alteration to executive functions [15,16], mood [16], and domestic accidents in older people living in the community [17]. In the non-institutionalized elderly, the association of sociodemographic factors, cognitive status, and self-reported health has been investigated [18].

Mild cognitive impairment (MCI) is a heterogeneous clinical state in which cognitive changes evaluated over time can evolve towards dementia, remain stable, or go back to normal [19,20]. These changes can be modulated by clinical (diabetes, depression, high blood pressure), demographic (old age, female gender, low level of education), and occupational (low level of mental occupational state) [21,22] variables and are associated with a lower Mini-Mental State Examination (MMSE) score, a higher risk of dementia, and increased mortality [23,24,25].

Neuropsychological evaluations monitor these changes and help to select subgroups as possible objectives for prevention or therapeutic interventions [4,5,6,26]. Some studies identified the efficiency of these interventions in reducing cognitive impairment as a knowledge gap [27]. Some recent publications point out the need to identify the study population’s individual characteristics and its initial cognitive impairment to design more efficient personalized interventions [28,29].

This study aimed to determine the differences and cognitive predictors for each gender in older adults with SMCs who live in the general community and are not institutionalized to set up preventive programs in the future.

We hypothesized that men with a higher level of education and a higher occupational level would have better global cognitive capacity, particularly in the calculation and attention domains, whereas women who have more cardiovascular (CV) risk factors would display worse cognitive performance and would stand out for their verbal fluency. Our hypothesis is supported in the literature by cognitive reserve models in aging, which suggest that the individual’s life experience (education, work activity, and leisure) may exert a neuroprotective effect against cognitive deterioration and may represent a major contribution to successful aging [30,31].

## 2. Subjects and Methods

This descriptive observational study was conducted in a primary healthcare center in the Zaragoza province (NE Spain). The sample was made up of 367 participants with SMCs who attended primary healthcare clinics and received the usual therapy and nursing care.

The participants received information about the project from informative posters placed on the doors of all the medical consultation rooms and where their family doctors worked.

The sample size calculation was performed with the 95% confidence level and a 5% sampling error. The final evaluated sample was representative of the Zaragoza province.

The inclusion criteria were as follows: ≥65 years with the Spanish version of Mini-Mental State Examination (MEC-35) [32] score between 24 and 35 points suffering from SMCs. The exclusion criteria were as follows: institutionalization and cognitive stimulation in the past 12 months.

The following sociodemographic variables were studied: gender, age, level of education, civil status, and mental occupational, physical occupational, and clinical states, such as high blood pressure (HBP), diabetes, hypercholesterolemia, obesity, and cerebrovascular accidents (CVA). Moreover, an analysis of the subgroups was considered according to the level of education (primary/higher), physical occupational status and mental occupational status based on three levels: low, medium, and high (for each) [33].

### 2.1. Neuropsychological Assessment

The existence of SMCs was evaluated with the question “Do you have complaints about your memory?” (dichotomous response (yes/no)) [34,35].

The primary variable was MEC-35, which is considered one of the most widely used short cognitive tests to study cognitive capacities in primary care. It evaluates eight components: spatiotemporal orientation (10 points), fixation memory (3 points), attention (3 points), calculation (5 points), short-term memory (3 points), language and praxis (11 points). Its sensitivity is 85–90% and its specificity is 69%. With this questionnaire, global cognition and cognitive functions were evaluated. Classification is based on scores: 30–35 points for people considered to have normal cognitive function; 25–29 points for borderline cognitive deficits; 20–24 points for MCI; 15–19 points for moderate cognitive impairment; ≤14 points for severe cognitive impairment [32]. Unlike the MMSE, MEC-35 includes a three-digit series to repeat two similar items in the reverse order, and subtraction is performed by subtracting three from 30 instead of seven from 100, as in the version of Folstein et al. As the number of items increases, the maximum score in this version reaches 35 points compared to 30 in the original one [36].

We considered using the Spanish version of the MMSE (MEC-35) to assess global cognition and to observe if there was any change in cognitive functions. Other authors warrant further investigation if the overall MMSE assessment reveals areas of concern [37]. Gómez Gallego et al. mentioned that the MMSE allows the rapid assessment of cognitive functions and considering the functions of different domains [38]. The validity data of the individual MEC-35 items are also satisfactory (particularly with temporal orientation) [32]. In Spain, it is common to resort to the adaptation of the MMSE proposed by Lobo et al. in 1979 [39] called MEC-35, because some items of the original version of Folstein are difficult for patients with a low cultural level, which affects the scale’s discriminative capacity [36].

The secondary variable was the set test [40] which measures category verbal fluency: colors, animals, fruits, cities. Its sensitivity is 87% and its specificity is 67%.

The evaluation process was performed by occupational therapists after receiving the corresponding training to guarantee homogeneous application of evaluation instruments.

### 2.2. Statistical Analysis

The statistical analysis was performed with the IBM SPSS Statistics Package, v.22. (SPSS Inc., Chicago, IL, USA). The descriptive statistics are shown according to the nature of each variable: mean (m) and standard deviation (SD) or the number of participants in each category (n) and the proportion of patients in relation to the total (%). The normality of the variables was verified by Kolmogorov–Smirnov test.

To analyze cognitive characteristics for each gender, the non-parametric Mann–Whitney U-test was used. To measure cognitive characteristics according to the mental occupation state, Kruskal–Wallis H-test was applied. The level of significance was set at 5%.

### 2.3. Ethical Considerations

This study was approved by the Research Ethics Committee of the Spanish Autonomous Community of Aragón, protocol number (CEICA PI11/90). Personal data protection regulations were respected. All the participants were informed about the study objectives and signed informed consent. The deontological norms recognized by the Declaration of Helsinki (52nd World Medical Association (WMA) General Assembly, Edinburgh, Scotland, October 2000) [41] and good clinical practice norms were followed, and current legislation was complied with.

## 3. Results

This study included 367 older adults with MEC-35 scores between 24 and 35 points; 66.48% (244) were women and 33.51% (123) were men. Their mean age was 73.85 years, with the SD of 5.99. Table 1 lists their sociodemographic and clinical characteristics.

Regarding the sociodemographic variables, for level of education, 76.8% stated primary education, and 23.2%—higher education. Regarding civil status, 67% were married, 24.5% were widowed, 5.2% were single, and 3.3% were separated. Occupational physical status was low for 20.2%, medium for 43.6%, and high for 36.2% of the cases. Mental occupational status was low for 59.1% of the cases, medium for 35.1%, and high for 5.7%. Except for age (*p* = 0.188), all the other variables were statistically significant and favored men (*p* < 0.001).

The most diagnosed CV disease was HBP (48.8%), followed by hypercholesterolemia (37.9%), diabetes (14.2%), obesity (13.6%), and CVA (6.5%). The gender study revealed that diabetes and CVA were more frequent in men, as were hypercholesterolemia and obesity in women. These differences were statistically significant (*p* < 0.001). HBP was more frequent in men, but not significantly so (*p* = 0.639).

Table 2 shows the comparative study of the cognitive variables. For the primary variable MEC-35, the mean for the overall level was higher for men and statistically significant (*p* **<** 0.001). When analyzing the different MEC-35 domains, higher levels were obtained for attention, calculation, and language for men, with statistically significant differences for women (*p* < 0.001, *p* < 0.001, and *p* = 0.013, respectively). The verbal fluency cognitive set test variable was slightly higher in women, but the difference was not statistically significant (*p* = 0.105).

Table 3 shows the differential cognitive characteristics for mental occupational status (low, medium, high), where the global MEC-35 was higher in the older adults with a high mental occupational status, which was statistically significant (*p* < 0.001). Regarding the MEC-35 domains, temporal orientation (*p* = 0.034), attention (*p* < 0.001), calculation (*p* = 0.003), and language (*p* < 0.001) were higher in the older adults with a high mental occupational status, and these differences were statistically significant. However, with verbal fluency which was measured by the set test, the score was higher for the elderly with a higher mental occupational status. This difference was statistically significant.

## 4. Discussion

This study explored the cognitive profile of the elderly population who attended the Occupational Therapy area of a primary healthcare center in Spain in, and it compared gender and occupational differences. These results have scarcely been reported in previous studies with a view to subsequently design personalized therapeutic interventions that adapt to older adults’ initial cognitive performance and previous mental/physical occupational status. The factors analyzed herein covered sociodemographic factors, CV factors, and mental/physical occupational status factors [21,22,23,24,25].

Some of these factors have not been jointly explored in the past. The analyzed data of the cognitive clinical variables were disaggregated in the various cognitive domains and compared per gender. Verbal fluency was studied using the set test tool, which is apt for people who are illiterate or have a low level of education. The analyzed occupational cognitive variables were also studied per gender [32,40].

This study addressed the research gap related to a previous therapeutic analysis, which should be performed before designing and applying any type of cognitive stimulation therapy with elderly populations living in the general community to prevent and revert cognitive impairment [27]. The gender differences that the present study identified could help to design personalized and adapted interventions in more efficient occupational and cognitive terms [28,29].

Despite fewer men participating in this study and them being older, with which the literature associates a higher cognitive impairment risk [21,42,43,44,45] and a lower MMSE cognitive score [46], the detailed analysis of the other sociodemographic characteristics allowed us to determine that the male gender is protected by other factors like having higher levels of education, mental occupational status, and physical occupational status than women, as well as most men being married.

Despite the fact that men were older than women, there were no statistically significant differences for age. As in our study, Liu et al. [47] found no significant differences in cognitive impairment for age or gender until the age of 75 years. Likewise, studies into dementia prevalence have concluded that before reaching the age of 75 years, this rate is similar between men and women, but this prevalence is significantly higher for women aged more than 75 years [44]. Although older women are at a significantly higher risk for cognitive impairment than men [48], the women in our study were younger.

In our study, women reported a lower level of education than men. Education and literacy explain 60% of female disadvantage in cognitive functioning for the main effect of being women [49] This is consistent with previous findings indicating that a low level of education is associated with not only greater cognitive impairment and dementia [42,43], but also with worse initial cognitive performance [18]. Being female and having a lower level of education are considered to act as additional risks of cognitive impairment [44,48]. Other authors have, however, found no gender differences in cognitive dysfunctions [43]. From a public health perspective, it may be more efficacious to increase levels of education and improve access to resources than to intervene in chronic diseases that are closely linked with cognitive deterioration outcome [50].

Regarding civil status, we identified a higher percentage of married people, especially men, although the percentage of married women exceeded 50%. If we consider that women live longer, a higher proportion of widowed women is justified. The civil status relation with cognitive functioning is based on the marital resources model. This model explains that being married is associated with unique social, psychological, and economic resources that promote health and longevity in the elderly by using compensatory cognitive approaches that increase neuronal plasticity and improve the cognitive reserve [51]. Conversely, divorced, single, or widowed older adults are more vulnerable to cognitive impairment because they are more likely to present alterations to different cognitive domains [47] and develop dementia than married people [52].

Regarding the CV risk factors, our study found that diabetes and CVA were more frequent in men. However, cholesterol and obesity rate were higher in women. Although HBP was more frequent in men, it was not significantly so. When we compared our data to the cross-sectional study by Niu et al. [53], more older adults in China presented diabetes (18% vs. our 14.2%), hypercholesterolemia (40.2% vs. our 37.9%), and HBP (51.9% vs. 48.8%), but less adults presented obesity (10.8% vs. our 13.6%).

For arterial hypertension, no other studies found similar results, with a higher prevalence in women in India (3.4% in men, 6.8% in women) and Poland (68.9% in men, 72.5% in women) [54]. In the study by Al-Nozha et al. [55], the percentage of women with systolic hypertension (15.7%) was significantly higher than that of men (11.0%) (*p* < 0.01). On the contrary, other authors reported a higher prevalence in men [56]. Hypertension is an important risk factor for vascular dementia and Alzheimer’s disease [57]. Poor blood pressure (BP) control is associated with an even greater cognitive decline [58]. Other studies indicate a higher prevalence in women in relation to hypercholesteremia (60% of men versus 68% of women) without any statistically significant differences like in our study [59]. Other studies found, like us, a higher prevalence of obesity in women [60,61,62]. Although our study did not find high CV disease figures for women, it is considered the main cause of morbimortality in post-menopausal women [63]. CV disease is generally associated with not only worse cognitive functioning in executive skills, processing speed, verbal memory, and attention [64,65], but also with a high rate of SMCs [66]. In any case, most CV risk factors are potentially preventable or treatable [67].

When examining the cognitive variables in this study, we found that men presented a higher basal global cognitive level than women, as measured by MEC-35. This higher cognitive functioning level was possibly modulated by the cited sociodemographic variables (higher level of education, higher mental occupational status). The domains analysis indicated larger statistically significant differences for attention, calculation, and language for men than for women.

In cognitive terms, the gender differences for initial cognitive performance were not conclusive. We identified studies that report higher mean cognitive scores for women [18], although most studies support our results because they indicate that those global cognitive differences favoring the male gender appear at different aging stages in normal cognition, MCI, and dementia [67,68,69]. Therefore, longitudinal analyses should study the mechanism by which this greater cognitive impairment in women is determined [70], which acts as a predictor of mortality in the follow-up studies performed with older adults living in the general community [44].

Gender stereotypes maintain that men surpass women in calculation and spatial tests, while women do better in verbal tests than men [71]. As for men better performing in the calculation domain, this could be due to them reporting a higher level of education. Along these lines, the literature indicates that older adults with a higher level of education perform arithmetic tasks better because they have access to previously acquired knowledge of reasoning concepts and do not depend on executive and computational tasks, which also enhances their cognitive reserve [25,72].

Our study found that the cognitive attention/calculation domain values were higher for men. Verbal fluency, however, was higher for women, but not significantly so. Therefore, our results are not conclusive. Some studies explain these gender differences by hemispheric asymmetry [73], while other authors conclude that this asymmetry would not cause such differences in cognitive functioning between men and women [74]. It is necessary to stress that attention is one of the most vulnerable domains to cognitive impairment, while verbal capacities are affected later [6]. More engagement in self-improvement for men was related to a higher level of cognitive functioning. For women, intellectual–cultural activity has been related to better verbal ability and memory. Concerning cognitive function evolution, domestic activity among men has been related to a less marked decline in speed, whereas for women, it has been associated with a more marked decline in spatial ability and memory. Furthermore, women’s greater intellectual–cultural activity has been related to a more significant decline in memory [75].

When analyzing the occupational elements, our study revealed that men developed mental capacities when they worked better than women. Similar results were reported by other authors, who demonstrated that those occupations involving higher mental demands enhance cognitive functioning, which may come over as better cognitive performance after retirement in older adults [21,33]. Mental demand has been considered a factor that protects older adults from functional loss [76]. In addition, memory, learning, attention, and information processing are influenced by the kind of work and aging itself, which can be reversed by developing competences to perform work [77]. Li et al. evaluated the association between the principal lifetime occupation and risk of cognitive impairment in older adults and found that occupations with a low level of mental demand were associated with greater cognitive impairment [78]. It appears that protective effects derive from continuous lifelong cognitive stimulation, including formal education, participation in cognitive stimulation activities, and occupation, which protect against age-related cognitive decline and reduce the risk of developing Alzheimer’s disease [79]. According to Min et al., longer working hours, especially for men and manual workers, appear to be a significant contributor to cognitive and physical function in older people [80].

This study is not without its limitations. First, the measurement of cognitive impairment is based on cognitive tests and could be subject to measurement errors. However, cognitive impairment was identified herein using different MEC-35 cut-off points and a suitable supplementary verbal fluency tool for illiterate people. Second, the use of the Spanish version of the MMSE to assess cognitive functions without adding other complementary tools. Third, the participants were recruited from a primary healthcare center in a specific neighborhood and not randomly drawn from the community. Therefore, we do not know if the sociodemographic parameters would coincide, extrapolate, and generalize to other population groups. Finally, the fact that occupational therapists do not form part of public health services in health centers is a limitation for the participants to understand the study objectives.

As future research lines, multicenter observational studies with larger sample sizes are necessary to more accurately determine gender predictors of cognitive impairment to examine other factors not considered herein, such as performing activities of daily living and psychological factors like anxiety and depression. Another aspect to take into account is that verbal memory improves in cognitive training programs and an improvement in executive functions has been observed in people with subjective memory in cognitive stimulation programs, while improvements in cognition have generally been noted in both [81].

## 5. Conclusions

The conclusion drawn from the study is that some personalized interventions could be developed, such as cognitive training, which may improve cognitive functioning, prevent, slow down, and revert adverse effects of cognitive aging [4] and contemplate certain sociodemographic and clinical variables per gender to successfully perform challenging activities that increase cerebral neuroplasticity and create deeper learning curves in our older Spanish adults.

Furthermore, the strong point of this study is that it can be generally applied to therapeutic intervention practice. Having evaluated older adults’ occupational characteristics and previous level of education and not only their basal cognitive performance, but also cognitive domains, will act as a guidance for occupational therapists to design more personalized interventions that adapt to these characteristics.

## Figures and Tables

**Table 1:** The patients’ sociodemographic and clinical characteristics per gender.

	Total (*n* = 367)	Men (*n* = 123)	Women (*n* = 244)	*p*-Value
Age (Years)	Mean ± SD 73.85 ± 5.99	Mean ± SD/% 74.40 ± 5.93	Mean ± SD/% 73.57 ± 6.00	0.188
	*n* (%)	*n* (%)	*n* (%)	
**Level of education**				
Primary	282 (76.8%)	82 (66.7%)	200 (82%)	<0.001 **
Higher	85 (23.2%)	41 (33.3%)	44 (18%)	
**Civil status**				
Single	(5.2%)	(5.1%)	(5.3%)	<0.001 **
Married	(67%)	(73.6%)	(59.4%)	
Widowed	(24.5%)	(16.8%)	(33.5%)	
Separated	(3.3%)	(4.6%)	(1.8%)	
**Physical occupational**				
Low	74 (20.2%)	26 (21.1%)	48 (19.7%)	<0.001 **
Medium	160 (43.6%)	40 (32.5%)	120 (49.2)	
High	133 (36.2%)	57 (46.3%)	76 (31.1%)	
**Mental occupational**				
Low	217 (59.1%)	48 (39%)	169 (69.3%)	<0.001 **
Medium	129 (35.1%)	59 (48%)	70 (28.7%)	
High	21 (5.7%)	16 (13.2%)	5 (2%)	
**HBP**				
Yes	179 (48.8%)	63 (51.2%)	116 (47.5%)	0.639
No	188 (51.2%)	60 (48.8%)	128 (52.5%)	
**Diabetes**				
Yes	52 (14.2%)	22 (17.9%)	30 (12.3%)	<0.001 **
No	315 (85.8%)	101 (82.1%)	214 (87.7%)	
**Hypercholesterolemia**				
Yes	139 (37.9%)	41 (33.3%)	98 (40.2%)	<0.001 **
No	228 (62.1%)	82 (66.7%)	146 (59.8%)	
**Obesity**				
Yes	50 (13.6%)	16 (13%)	34 (13.9%)	<0.001 **
No	317 (86.4%)	107 (87%)	210 (86.1%)	
**CVA**				
Yes	24 (6.5%)	10 (8.1%)	14 (5.7%)	<0.001 **
No	343 (93.5%)	113 (91.9%)	230 (94.3%)	

CVA: cerebrovascular accident; *p*-value: Pearson’s Chi-squared test value. ** *p* < 0.001.

**Table 2:** Differential cognitive characteristics per gender (mean + standard deviation).

	Gender Study			
	Total (*n* = 367)	Men (*n* = 123)	Women (*n* = 244)	*p*-Value
Global MEC-35	29.48 (3.18)	30.37 (3.08)	29.48 (3.15)	<0.001 **
Temporal orientation	4.39 (0.92)	4.38 (0.95)	4.40 (0.91)	0.968
Spatial orientation	4.66 (0.62)	4.67 (0.63)	4.65 (0.61)	0.570
Fixation memory	3.00 (0.52)	3.00 (0.00)	3.00 (0.06)	0.478
Attention	1.74 (1.22)	2.11 (1.18)	1.56 (1.20)	<0.001 **
Calculation	4.38 (1.00)	4.74 (0.71)	4.19 (1.08)	<0.001 **
Short-term memory	1.60 (1.09)	1.53 (1.14)	1.63 (1.07)	0.456
Language	5.28 (0.84)	5.43 (0.78)	5.21 (0.86)	0.013 *
Praxis	4.42 (0.70)	4.51 (0.65)	4.37 (0.72)	0.073
Set test	37.34 (3.79)	37.08 (3.73)	37.46 (3.88)	0.105

MEC-35: Spanish version of the Mini-Mental State Examination; *p*-value: Mann–Whitney U-test. * *p* < 0,05, ** *p* < 0.001.

**Table 3:** Differential cognitive characteristics per mental occupational status (mean + standard deviation).

	Mental Occupational Status Study				*p*-Value
	Total (*n* = 367)	Low (*n* = 197)	Medium (*n* = 170)	High (*n* = 170)	
Global MEC-35	29.48 (3.18)	28.80 (3.02)	30.28 (3.19)	31.52 (2.87)	<0.001 **
Temporal orientation	4.39 (0.92)	4.33 (0.94)	4.47 (0.85)	4.62 (1.12)	0.034 *
Spatial orientation	4.66 (0.62)	4.63 (0.63)	4.70 (0.59)	4.71 (0.56)	0.577
Fixation memory	3.00 (0.52)	3.00 (0.00)	2.99 (0.09)	3.00 (0.00)	0.398
Attention	1.74 (1.22)	1.53 (1.20)	2.03 (1.18)	2.24 (1.14)	<0.001 **
Calculation	4.38 (1.00)	4.27 (1.05)	4.48 (0.97)	4.86 (0.36)	0.003 *
Short-term memory	1.60 (1.09)	1.53 (1.09)	1.68 (1.11)	1.76 (1.04)	0.334
Language	5.28 (0.84)	5.16 (0.85)	5.42 (0.84)	5.71 (0.46)	<0.001 **
Praxis	4.42 (0.70)	4.35 (0.76)	4.50 (0.60)	4.62 (0.59)	0.105
Set test	37.34 (3.79)	36.77 (4.13)	38.14 (3.17)	38.24 (2.36)	0.002 *

MEC-35: Spanish version of the Mini-Mental State Examination; *p*-value: Kruskal–Wallis H-test. * *p* < 0,05, ** *p* < 0.001.

## Data Availability

The data presented in this study are available upon request from the corresponding author.

## References

[1] Toricelli M., Pereira A.A.R., Souza Abrao G., Malerba H.N., Maia J., Buck H.S., Viel T.A. (2021). Mechanisms of neuroplasticity and brain degeneration: Strategies for protection during the aging process. Neural Regen. Res..

[2] Organización Mundial de la Salud 2015 [Internet] Ginebra: Informe Mundial Sobre el Envejecimiento y la Salud. Informe de un Grupo Científico de la OMS. Ginebra; 2015. https://apps.who.int/iris/bitstream/handle/10665/186466/9789240694873_spa.pdf;jsess%20ionid=A18FC62E4436A9287985DD570C9D687F?sequence=1.

[3] Silva E., Farias I.P., Montenegro L.A.S., Wanderley R.L., de Pontes J.C.X., Pereira A.C., de Almeida L.D.F.D., Cavalcanti Y.W. (2020). Physical and psychological states interfere with health-related quality of life of institutionalized elderly: A cross-sectional study. BMC Geriatr..

[4] Chen S.T., Volle D., Jalil J., Wu P., Small G.W. (2019). Health-Promoting Strategies for the Aging Brain. Am. J. Geriatr. Psychiatry..

[5] Dumas J.A. (2017). Strategies for Preventing Cognitive Decline in Healthy Older Adults. Can. J. Psychiatry.

[6] Cohen R.A., Marsiske M.M., Smith G.E. (2019). Neuropsychology of aging. Handb. Clin. Neurol..

[7] Salthouse T.A. (2018). Why is cognitive change more negative with increased age?. Neuropsychology.

[8] Mora-Gallegosa A., Salas S., Fornaguera-Tríasa J. (2017). Efectos del enriquecimiento ambiental dependiente de la edad en el comportamiento, funciones cognitivas y neuroquímica. Rev. Mex. Neurocienc..

[9] Basak C., Qin S., O’Connell M.A. (2020). Differential effects of cognitive training modules in healthy aging and mild cognitive impairment: A comprehensive meta-analysis of randomized controlled trials. Psychol. Aging.

[10] Maass A., Lockhart S.N., Harrison T.M., Bell R.K., Mellinger T., Swinnerton K., Baker S.L., Jagust W.J. (2018). Entorhinal Tau Pathology, Episodic Memory Decline, and Neurodegeneration in Aging. J. Neurosci..

[11] Jessen F., Amariglio R.E., van Boxtel M., Breteler M., Ceccaldi M., Chételat G., Subjective Cognitive Decline Initiative (SCD-I) Working Group (2014). A conceptual framework for research on subjective cognitive decline in preclinical Alzheimer’s disease. Alzheimers Dement..

[12] Mitchell A.J., Beaumont H., Ferguson D., Yadegarfar M., Stubbs B. (2014). Risk of dementia and mild cognitive impairment in older people with subjective memory complaints: Metaanalysis. Acta Psychiatr. Scand..

[13] Tsutsumimoto K., Makizako H., Doi T., Hotta R., Nakakubo S., Makino K., Suzuki T. (2017). Subjective Memory Complaints are Associated with Incident Dementia in Cognitively Intact Older People, but Not in Those with Cognitive Impairment: A 24-Month Prospective Cohort Study. Am. J. Geriatr. Psychiatry.

[14] Takechi H., Tsuzuki A., Matsumoto K., Matsunaga S., Nishiyama H., Ogawa M., Kanada Y. (2020). Relationship between subjective memory complaints and social and leisure activities in community-dwelling older people: Toyoake Integrated Care Study. Geriatr. Gerontol. Int..

[15] Cespón J., Galdo-Álvarez S., Díaz F. (2018). Event-Related Potentials Reveal Altered Executive Control Activity in Healthy Elderly with Subjective Memory Complaints. Front. Hum. Neurosci..

[16] Spano G., Caffò A.O., Lanciano T., Curci A., Bosco A. (2020). Visuospatial/executive abilities and mood affect the reliability of a subjective memory complaints measure. Aging Clin. Exp. Res..

[17] Spano G.O., Caffò A., Bosco A. (2018). Cognitive functioning, subjective memory complaints and risky behaviour predict minor home injuries in elderly. Aging Clin. Exp. Res..

[18] Brody D.J., Kramarow E.A., Taylor C.A., McGuire L.C. (2019). Cognitive Performance in Adults Aged 60 and Over: National Health and Nutrition Examination Survey, 2011–2014. Natl. Health Stat. Rep..

[19] Pierce S., Lamers C., Salisbury K. (2016). Knowingly not wanting to know: Discourses of people diagnosed with mild cognitive impairment. Dementia.

[20] Song X., Mitnitski A., Zhang N., Chen W., Rockwood K. (2013). Alzheimer’s Disease Neuroimaging Initiative. Dynamics of brain structure and cognitive function in the Alzheimer’s disease neuroimaging initiative. J. Neurol. Neurosurg. Psychiatry.

[21] Chung W., Kim R. (2020). Differential Risk of Cognitive Impairment across Paid and Unpaid Occupations in the Middle-Age Population: Evidence from the Korean Longitudinal Study of Aging, 2006–2016. Int. J. Environ. Res. Public Health.

[22] Yaffe K. (2018). Modifiable Risk Factors and Prevention of Dementia: What Is the Latest Evidence?. JAMA Intern. Med..

[23] Li J.Q., Tan L., Wang H.F., Tan M.S., Tan L., Xu W., Zhao Q.F., Wang J., Jiang T., Yu J.T. (2016). Risk factors for predicting progression from mild cognitive impairment to Alzheimer’s disease: A systematic review and meta-analysis of cohort studies. J. Neurol. Neurosurg. Psychiatry.

[24] Wang M.C., Li T.C., Li C.I., Liu C.S., Lin C.H., Lin W.Y., Lin C.C. (2020). Cognitive function and its transitions in predicting all-cause mortality among urban community-dwelling older adults. BMC Psychiatry.

[25] Zamarian L., Lenhart L., Nagele M., Steiger R., Gizewski E.R., Benke T., Delazer M. (2020). Effects of Cognitive Functioning and Education on Later-Life Health Numeracy. Gerontology.

[26] Di Carlo A., Baldereschi M., Lamassa M., Bovis F., Inzitari M., Solfrizzi V., Inzitari D. (2016). Italian Longitudinal Study on Aging Working Group. Daily Function as Predictor of Dementia in Cognitive Impairment, No Dementia (CIND) and Mild Cognitive Impairment (MCI): An 8-Year Follow-Up in the ILSA Study. J. Alzheimers Dis..

[27] Smart C.M., Karr J.E., Areshenkoff C.N., Rabin L.A., Hudon C., Gates N., Subjective Cognitive Decline Initiative (2017). Non-Pharmacologic Interventions for Older Adults with Subjective Cognitive Decline: Systematic Review, Meta-Analysis, and Preliminary Recommendations. Neuropsychol. Rev..

[28] Teixeira-Santos A.C., Moreira C.S., Magalhães R., Magalhães C., Pereira D.R., Leite J., Sampaio A. (2019). Reviewing working memory training gains in healthy older adults: A meta-analytic review of transfer for cognitive outcomes. Neurosci. Biobehav. Rev..

[29] Matysiak O., Kroemeke A., Brzezicka A. (2019). Working Memory Capacity as a Predictor of Cognitive Training Efficacy in the Elderly Population. Front. Aging Neurosci..

[30] Stern Y. (2009). Cognitive reserve. Neuropsychologia.

[31] Caffò A.O., Lopez A., Spano G., Saracino G., Stasolla F., Ciriello G., Grattagliano I., Lancioni G.E., Bosco A. (2016). The role of pre-morbid intelligence and cognitive reserve in predicting cognitive efficiency in a sample of Italian elderly. Aging Clin. Exp. Res..

[32] Lobo A., Saz P., Marcos G., Día J.L., Cámara C., de la Ventura T., Aznar S. (1999). Revalidación y normalización del Mini-Examen Cognoscitivo (primera versión en castellano del Mini-Mental Status Examination) en la población general geriátrica. Med. Clin..

[33] Grotz C., Meillon C., Amieva H., Andel R., Dartigues J.F., Adam S., Letenneur L. (2018). Occupational social and mental stimulation and cognitive decline with advancing age. Age Ageing.

[34] Gagnon M., Dartigues J.F., Mazaux J.M., Dequae L., Letenneur L., Giroire J.M., Barberger-Gateau P. (1994). Self-reported memory complaints and memory performance in elderly French community residents: Results of the PAQUID Research Program. Neuroepidemiology.

[35] Van Oijen M., de Jong F.J., Hofman A., Koudstaal P.J., Breteler M.M. (2007). Subjective memory complaints, education, and risk of Alzheimer’s disease. Alzheimers Dement..

[36] Calero M.D., Navarro E., Robles P., García-Berbén T.M. (2000). Estudio de validez del Mini-Examen Cognoscitivo de Lobo et al. para la detección del deterioro cognitivo asociado a demencias. Neurología.

[37] Norris D., Clark M.S., Shipley S. (2016). The Mental Status Examination. Am. Fam. Physician.

[38] Gómez Gallego M., Gómez García J. (2017). Musicoterapia en la enfermedad de Alzheimer: Efectos cognitivos, psicológicos y conductuales. Neurología.

[39] Lobo A., Escobar V., Ezquerra J., Seva Díaz A. (1979). El Mini-Examen Cognoscitivo: Un test sencillo, práctico, para detectar alteraciones intelectuales en pacientes psiquiátricos. Actas Luso-Españolas Neurol. Psiquiatr..

[40] Pascual L.F., Martínez J.V., Modrego P., Mostacero E., López del Val J., Morales F. (1990). El Set-test en el diagnóstico de la demencia. Neurología.

[41] Declaración de Helsinki de la AMM Principios éticos Para las Investigaciones Médicas en Seres Humanos [Internet]. 52a Asamblea General de la AMM, Edimburgo, Escocia: Asociación Médica Mundial; 2020. https://www.wma.net/wp-content/uploads/2018/07/DoH-Oct2000.pdf.

[42] Sharifi F., Fakhrzadeh H., Varmaghani M., Arzaghi S.M., Alizadeh Khoei M., Farzadfar F., Tanjani P.T. (2016). Prevalence of Dementia and Associated Factors among Older Adults in Iran: National Elderly Health Survey (NEHS). Arch. Iran. Med..

[43] Warren L.A., Shi Q., Young K., Borenstein A., Martiniuk A. (2015). Prevalence and incidence of dementia among indigenous populations: A systematic review. Int. Psychogeriatr..

[44] Wang J., Xiao L.D., Wang K., Luo Y., Li X. (2020). Cognitive Impairment and Associated Factors in Rural Elderly in North China. J. Alzheimers Dis..

[45] Tsang S., Sperling S.A., Park M.H., Helenius I.M., Williams I.C., Manning C. (2017). Blood Pressure Variability and Cognitive Function Among Older African Americans: Introducing a New Blood Pressure Variability Measure. Cogn. Behav. Neurol..

[46] Nunes B., Silva R.D., Cruz V.T., Roriz J.M., Pais J., Silva M.C. (2010). Prevalence and pattern of cognitive impairment in rural and urban populations from Northern Portugal. BMC Neurol..

[47] Liu H., Zhang Y., Burgard S.A., Needham B.L. (2019). Marital status and cognitive impairment in the United States: Evidence from the National Health and Aging Trends Study. Ann. Epidemiol..

[48] Miyawaki C.E., Liu M. (2019). Gender differences in cognitive impairment among the old and the oldest-old in China. Geriatr. Gerontol. Int..

[49] Lee J., Shih R., Feeney K., Langa K.M. (2014). Gender disparity in late-life cognitive functioning in India: Findings from the longitudinal aging study in India. J. Gerontol. B Psychol. Sci. Soc. Sci..

[50] Alvarado B.E., Zunzunegui M.V., Del Ser T., Béland F. (2002). Cognitive decline is related to education and occupation in a Spanish elderly cohort. Aging Clin. Exp. Res..

[51] Sommerlad A., Ruegger J., Singh-Manoux A., Lewis G., Livingston G. (2018). Marriage and risk of dementia: Systematic review and meta-analysis of observational studies. J. Neurol. Neurosurg. Psychiatry.

[52] Liu H., Zhang Z., Choi S.W., Langa K.M. (2020). Marital Status and Dementia: Evidence from the Health and Retirement Study. J. Geontol. B Psychol. Sci. Soc. Sci..

[53] Ni W., Weng R., Yuan X., Lv D., Song J., Chi H., Xu J. (2019). Clustering of cardiovascular disease biological risk factors among older adults in Shenzhen City, China: A cross-sectional study. BMJ Open.

[54] Kearney P.M., Whelton M., Reynolds K., Whelton P.K., He J. (2004). Worldwide prevalence of hypertension: A systematic review. J. Hypertens..

[55] Al-Nozha M.M., Ali M.S., Osman A.K. (1997). Arterial hypertension in Saudi Arabia. Ann. Saudi Med..

[56] Sharma S.K., Ghimire A., Radhakrishnan J., Thapa L., Shrestha N.R., Paudel N., Gurung K.R.M., Budathoki A., Baral N., Brodie D. (2011). Prevalence of hypertension, obesity, diabetes, and metabolic syndrome in Nepal. Int. J. Hypertens..

[57] Rosendorff C., Beeri M.S., Silverman J.M. (2007). Cardiovascular risk factors for Alzheimer’s disease. Am. J. Geriatr. Cardiol..

[58] Vinyoles E., De la Figuera M., Gonzalez-Segura D. (2008). Cognitive function and blood pressure control in hypertensive patients over 60 years of age: COGNIPRES study. Curr. Med. Res. Opin..

[59] Polychronopoulos E., Panagiotakos D.B., Polystipioti A. (2005). Diet, lifestyle factors and hypercholesterolemia in elderly men and women from Cyprus. Lipids Health Dis..

[60] Flegal K.M., Carroll M.D., Ogden C.L., Curtin L.R. (2010). Prevalence and trends in obesity among US adults, 1999–2008. JAMA.

[61] Arterburn D.E., Crane P.K., Sullivan S.D. (2004). The coming epidemic of obesity in elderly Americans. J. Am. Geriatr. Soc..

[62] Baranwal J.K., Maskey R., Sherchand O., Chaudhari R.K. (2018). Prevalence of obesity and its association with cvd risk factors in nepalese patients with type 2 diabetes. Eur. J. Biomed..

[63] Collins P., Webb C.M., de Villiers T.J., Stevenson J.C., Panay N., Baber R.J. (2016). Cardiovascular risk assessment in women—An update. Climacteric.

[64] Blumenthal J.A., Smith P.J., Mabe S., Hinderliter A., Welsh-Bohmer K., Browndyke J.N., Sherwood A. (2017). Lifestyle and Neurocognition in Older Adults with Cardiovascular Risk Factors and Cognitive Impairment. Psychosom. Med..

[65] Williams I.C., Park M.H., Tsang S., Sperling S.A., Manning C. (2018). Cognitive Function and Vascular Risk Factors Among Older African American Adults. J. Immigr. Minor. Health.

[66] Lourenco J., Serrano A., Santos-Silva A., Gomes M., Afonso C., Freitas P., Costa E. (2018). Cardiovascular Risk Factors Are Correlated with Low Cognitive Function among Older Adults Across Europe Based on The SHARE Database. Aging Dis..

[67] Heise V., Filippini N., Trachtenberg A.J., Suri S., Ebmeier K.P., Mackay C.E. (2014). Apolipoprotein E genotype, gender and age modulate connectivity of the hippocampus in healthy adults. Neuroimage.

[68] Riedel B.C., Thompson P.M., Brinton R.D. (2016). Age, APOE and sex: Triad of risk of Alzheimer’s disease. J. Steroid. Biochem. Mol. Biol..

[69] Pontifex M., Vauzour D., Minihane A.M. (2018). The effect of APOE genotype on Alzheimer’s disease risk is influenced by sex and docosahexaenoic acid status. Neurobiol. Aging..

[70] Gale S.D., Baxter L., Thompson J. (2016). Greater memory impairment in dementing females than males relative to sex-matched healthy controls. J. Clin. Exp. Neuropsychol..

[71] Hyde J.S. (2016). Sex and cognition: Gender and cognitive functions. Curr. Opin. Neurobiol..

[72] Fastame M.C., Melis S. (2020). Numeracy Skills, Cognitive Reserve, and Psychological Well-Being: What Relationship in Late Adult Lifespan?. Behav. Sci..

[73] English M.C.W., Maybery M.T., Visser T.A.W. (2020). Magnitude of sex differences in visual search varies with target eccentricity. Psychon. Bull. Rev..

[74] Hirnstein M., Hugdahl K., Hausmann M. (2019). Cognitive sex differences and hemispheric asymmetry: A critical review of 40 years of research. Laterality Asymmetries Body Brain Cogn..

[75] Hassing L.B. (2020). Gender Differences in the Association between Leisure Activity in Adulthood and Cognitive Function in Old Age: A Prospective Longitudinal Population-Based Study. J. Gerontol. B Psychol. Sci. Soc. Sci..

[76] Amorim J.S., Salla S., Trelha C.S. (2014). Factors associated with work ability in the elderly: Systematic review. Rev. Bras. Epidemiol..

[77] Martinez M.C., Latorre M.R.D.O., Fischer F.M. (2010). Capacidade para o trabalho: Revisão de literatura. Cien. SaudeColet..

[78] Li C.Y., Wu S.C., Sung F.C. (2002). Lifetime principal occupation and risk of cognitive impairment among the elderly. Ind. Health.

[79] Baldivia B., Andrade V.M., Bueno O.F.A. (2008). Contribution of education, occupation and cognitively stimulating activities to the formation of cognitive reserve. Dement. Neuropsychol..

[80] Min J.Y., Park J.B., Lee K.J., Min K.B. (2015). The impact of occupational experience on cognitive and physical functional status among older adults in a representative sample of Korean subjects. Ann. Occup. Environ. Med..

[81] Tsai A.Y., Yang M.J., Lan C.F., Chen C.S. (2008). Evaluation of effect of cognitive intervention programs for the community-dwelling elderly with subjective memory complaints. Int. J. Geriatr. Psychiatry.

